# Editorial: Lessons on T-Cells and Immune-Targeting Therapeutics in Coeliac Disease

**DOI:** 10.3389/fimmu.2021.756087

**Published:** 2021-09-03

**Authors:** Melinda Y. Hardy, Daniel Agardh, Robert P. Anderson

**Affiliations:** ^1^Immunology Division, Walter and Eliza Hall Institute of Medical Research, Parkville, VIC, Australia; ^2^Department of Medical Biology, University of Melbourne, Parkville, VIC, Australia; ^3^Unit of Diabetes and Celiac Disease, Department of Clinical Sciences, Lund University, Malmö, Sweden; ^4^Wesley Medical Research - The Wesley Hospital, Auchenflower, QLD, Australia

**Keywords:** autoimmunity, coeliac disease, dermatitis herpetiformis, prevention, T cells, transglutaminase, treatment

Moving forward from sole reliance on gluten-free diet to treat coeliac disease (CeD) requires consensus on the mechanism of gluten toxicity relevant to CeD. A more precise understanding of the role of gluten-reactive CD4+ T cells underpins innovative efforts to design targeted approaches to diagnose, treat, or prevent CeD. The field will also benefit from confirmatory studies that may provide useful road-maps for monitoring disease modulation during clinical trials. This Research Topic describes the current knowledge and highlights possible targets for new CeD treatment strategies.

## Insights Into CeD Immune Pathogenesis

Memory CD4+ T cells orchestrating and maintaining an adaptive immune response directed against a conserved set of selectively deamidated gluten peptides is broadly accepted as the primary cause for CeD. Dietary gluten provides the antigenic substrate for the immune response in CeD, but only after selective deamidation by host transglutaminases do CD4+ T cells efficiently recognise gluten. CD4+ T cells specific for gluten also support B cells and plasma cells specific for deamidated gluten peptides. While some highlight autoimmune aspects of CeD, these are limited to a humoral response to transglutaminase 2 associated with enteropathy, and also transglutaminase 3 and 6 in patients with skin and neurological complications, respectively. Humoral autoimmunity without an accompanying autoreactive CD4+ T-cell response has generally been explained by extracellular transglutaminases in host tissues serving as the carrier in classical “hapten-carrier” complexes, and immunogenic gluten peptides being the “hapten”. By virtue of their catalytic activity mediating transamidation, transglutaminases crosslink gluten peptides onto themselves. This allows transglutaminase-specific B cells to take up transglutaminase-gluten complexes, present antigenic gluten peptides, and receive help from gluten-specific CD4+ T cells. In clinical practice, exclusion of dietary gluten slowly reduces intestinal injury and is paralleled by falling levels and eventually disappearance of transglutaminase autoantibodies. CeD is driven by an environmental rather than self-antigen. Gluten and transglutaminase-gluten complexes being extracellular antigens is also the likely explanation for the preponderance of the CD4 subtype of T cells responding to gluten, and very few if any gluten-specific T cells of the CD8 subtype. Whether direct effects of gluten on innate immunity, non-immune cells, or structures such as tight junctions actually contribute to pathology in patients consuming gluten is an open question awaiting convincing, reproducible studies in patients to confirm *in vitro* findings. To date, therapeutics targeting innate immunity such as IL-15 and leaky tight junctions are yet to show clear benefit in CeD patients. In summary, the T cell-mediated adaptive immunity model serves well to explain the natural history, genetics, and reductionist immunological observations associated with CeD, and predicts that a variety of immune strategies may be effective for diagnosis, monitoring, treatment, and prevention of disease.

Voisine and Abadie provide a thorough review on the interplay between innate and adaptive immune compartments, gluten, tissue transglutaminase 2, and HLA in the development of CeD. They highlight that no separate element is sufficient to induce intestinal damage typical of CeD. They discuss use of their transgenic mouse model and highlight the importance of cytokines in these processes. This paper nicely ties together many elements of CeD pathogenesis and the topics published in this e-book.

The difficulty in isolating gluten-specific CD4+ T cells from small amounts of intestinal tissue means that the true proportion of CeD-specific pathogenic T cells in the gut is unclear. Qiao et al. report generating T cells clones by limiting dilution from duodenal tissue from six CeD patients with active disease (one refractory) and 3 controls. From 1652 T cell clones they observed gluten-reactivity in 24 (1.5%). A large proportion of T cell clones responded to whole protein but not to peptides containing previously described T cell epitopes (1), suggesting that undescribed epitopes still exist within gluten. However, due to pre-screening with wheat gluten in this study, the proportion of T cells reactive to peptides derived from barley, rye, or oats, or to additional sub-dominant gluten peptides recognised by circulating CD4+ T cells will need further investigation (2, 3).

CeD can present with a number of extra-intestinal manifestations, including skin and oral manifestations. Kemppainen et al. highlight the key differences in pathogenesis between CeD and the cutaneous form of CeD, dermatitis herpetiformis. They also discuss the gaps that remain in our understanding of immune cell subset involvement in the characteristic skin lesions. Sanchez-Solares et al. describe an increase in FoxP3+ cells within the oral mucosa of active and treated CeD patients. They suggest a role in tissue repair due to a correlation with altered epithelial integrity in CeD patients and increases in peripheral amphiregulin mRNA expression. These findings open the possibility for oral mucosal targeting during tolerogenic immunotherapy. Further studies are required to understand the immune milieu and functional capacity of cells within this tissue site in CeD.

## Biomarkers to Predict Development, Diagnose, and Monitor CeD

The advancement of new therapies for CeD is dependent on sensitive methods to detect changes in gluten-specific immune responses, as well as clinical responses. Smithson et al. describe potential biomarkers for CeD and refractory CeD in their review, and the utility of such biomarkers in measuring therapeutic intervention in CeD trials. They describe a number of different immune markers that should be considered.

Further to this, Hardy et al. describe the utility of a simple and highly sensitive whole blood cytokine release assay to monitor changes to the gluten-induced CD4+ T-cell response in CeD patients, which was then utilised for monitoring responses during the RESET CeD Phase 2 clinical trial testing a gluten-peptide immunotherapy (Nexvax2). The assay was effective on samples from patients without the need of a gluten challenge. They describe the optimal conditions for blood collection and highlight the promise of the assay in detecting attenuated gluten-peptide induced IL-2 and IFN-γ release following Nexvax2 treatment. An assay that does not require gluten challenge provides a useful tool for future CeD trials and following validation, could be used as a less invasive T cell-based CeD diagnostic.

Taavela et al. describe another useful aid in clinical trial monitoring and possibly in CeD diagnosis. They utilised APOA4 staining (a lipid-binding glycoprotein) on histological sections in CeD patients and controls, and showed a clear distinction of the villus-crypt border. Use of this marker increased the reliability and reproducibility of morphometrical villus height:crypt depth readings, in particular for hard to interpret samples where intestinal damage was present.

Rare gluten-specific T cells can be detected and enriched for using MHC class II tetramers (4), and may be useful to predict for CeD (5). Using tetramer-based cell sorting Dahal-Koirala et al. isolated CD4+ T cells specific for immunodominant gluten epitopes and performed a comprehensive analysis of the T cell receptor (TCR) sequences expressed in CeD patients. This is the largest TCR database of gluten-specific CD4+ T cells studied so far consisting of TCRs expressed by 3122 clonotypes from 63 CeD patients. They showed a large proportion expressed TRAV26-1:TRBV7-2 chains and the dominant CDR3 R-motif. Importantly, a consistent proportion of public TCRs were observed within different individuals. This points to the possibility of utilising TCR sequencing to support CeD diagnosis. Further validation studies are warranted.

Lastly, Auricchio et al. discuss biomarkers predictive for CeD development (e.g. microRNA’s and lipidomic modifications), and the possibility of disease prevention in at-risk individuals. They review prevention trials conducted to date and studies that target risk factors, such as childhood infections, changes in the microbiota, effects of vaccinations, gluten infant feeding and use of probiotics. They argue that successful prevention may require a combination in approaches.

## Conclusions

Together these articles highlight how understanding the contributions of CD4+ T cells in CeD pathogenesis is enabling clinical programs seeking to improve CeD clinical management or even prevent CeD altogether. Additional tools to detect disease processes are discussed, which will greatly benefit the field. As the adaptive immune paradigm strengthens, there could still be long-held beliefs about gluten toxicity in patients that are substantially revised. There are also a number of important unanswered questions, such as the exact triggers of CeD, the separation of the symptomatic response, gluten immunity, and the damaged intestine, the drivers of extra-intestinal manifestations, and if it is possible to prevent CeD in genetically at-risk populations. The next decade of research is set to provide new insights into CeD pathogenic processes, less invasive diagnostic tests, and better treatment options for CeD and refractory CeD patients with greater focus on targeting immune processes, and in particular halting the gluten-specific CD4+ T-cell response. Moreover, longitudinal follow-up of several ongoing primary and secondary prevention trials conducted in at-risk birth cohorts will be completed. Such insights will benefit researchers in the entire field of T cell-mediated disease.

## Author Contributions

All authors contributed to the article and approved the submitted version.

## Conflict of Interest

RA has served as consultant for Takeda, GSK, Anokion, Allero Therapeutics, Treg Therapeutics, EVOQ Therapeutics, and Bristol-Myers Squibb. He is also founder and shareholder of Novoviah Pharmaceuticals and is the inventor of patents relating to the diagnosis and treatment of coeliac disease.

The remaining authors declare that the research was conducted in the absence of any commercial or financial relationships that could be construed as a potential conflict of interest.

## Publisher’s Note

All claims expressed in this article are solely those of the authors and do not necessarily represent those of their affiliated organizations, or those of the publisher, the editors and the reviewers. Any product that may be evaluated in this article, or claim that may be made by its manufacturer, is not guaranteed or endorsed by the publisher.

## References

[1] Sollid LM Tye-Din JA Qiao SW Anderson RP Gianfrani C Koning F. Update 2020: Nomenclature and Listing of Celiac Disease-Relevant Gluten Epitopes Recognized by CD4(+) T Cells. Immunogenetics (2020) 72:85–8. 10.1007/s00251-019-01141-w 31735991

[2] Hardy MY Tye-Din JA Stewart JA Schmitz F Dudek NL Hanchapola I. Ingestion of Oats and Barley in Patients With Celiac Disease Mobilizes Cross-Reactive T Cells Activated by Avenin Peptides and Immuno-Dominant Hordein Peptides. J Autoimmun (2015) 56:56–65. 10.1016/j.jaut.2014.10.003 25457306

[3] Tye-Din JA Stewart JA Dromey JA Beissbarth T van Heel DA Tatham A. Comprehensive, Quantitative Mapping of T Cell Epitopes in Gluten in Celiac Disease. Sci Transl Med (2010) 2:41ra51. 10.1126/scitranslmed.3001012 20650871

[4] Christophersen A Raki M Bergseng E Lundin KE Jahnsen J Sollid LM. Tetramer-Visualized Gluten-Specific CD4+ T Cells in Blood as a Potential Diagnostic Marker for Coeliac Disease Without Oral Gluten Challenge. United European Gastroenterol J (2014) 2:268–78. 10.1177/2050640614540154 PMC4114117 25083284

[5] Sarna VK Lundin KEA Morkrid L Qiao SW Sollid LM Christophersen A. HLA-DQ-Gluten Tetramer Blood Test Accurately Identifies Patients With and Without Celiac Disease in Absence of Gluten Consumption. Gastroenterology (2018) 154:886–96.e6. 10.1053/j.gastro.2017.11.006 29146521

